# Clinical Manifestations, Tumor Associations, and Long-Term Outcomes of Anti-KLHL11 Encephalitis

**DOI:** 10.1212/NXI.0000000000200597

**Published:** 2026-05-29

**Authors:** Mengzhi Jin, Tessa Brand, Ece Erdag Turgeon, Alexander C. Havelaar, Robin W. Van Steenhoven, Mariska M.P. Nagtzaam, Suzanne C. Franken, Sarah H.B. Abu Hassan, Anna E.M. Bastiaansen, Juliette Brenner, Peter A.E. Sillevis Smitt, Esther de Graaff, Sharon Veenbergen, Juna M. de Vries, Jeroen Kerstens, Maarten J. Titulaer

**Affiliations:** 1 Department of Neurology, Erasmus University Medical Center, Rotterdam, the Netherlands;; 2 Department of Neurology, the First Affiliated Hospital of NanChang University, Jiangxi, China;; 3 Department of Neurology, National Neuroscience Institute, Singapore;; 4 Division of Cell Biology, Neurobiology and Biophysics, Department of Biology, Utrecht University, the Netherlands; and; 5 Department of Immunology, Laboratory Medical Immunology, Erasmus Medical Center, Rotterdam, the Netherlands.

## Abstract

**Background and Objectives:**

Anti–Kelch-like protein 11 (KLHL11) encephalitis was discovered in middle-aged men with testicular seminoma and rhombencephalitis, defining a new type of paraneoplastic neurologic syndrome (PNS), but diagnostic criteria and treatment outcomes remain largely unclear. This study aimed to comprehensively describe the initial presentations and subsequent clinical courses, ancillary findings, treatments, and outcomes of patients with anti-KLHL11 encephalitis.

**Methods:**

We tested 1,361 patients with clinical features or tumors that could be associated with anti-KLHL11 encephalitis (1,164 CSF and 680 serum). The retrospective analysis included 458 serum and 288 CSF samples from 473 patients between January 2010 and July 2020, while prospective screening for anti-KLHL11 antibodies was regularly performed since July 2020. Anti-KLHL11-abs was screened using KLHL11 overexpression cell-based assay. Detailed clinical and paraclinical information was collected.

**Results:**

Seventeen anti-KLHL11 encephalitis patients were identified. The median age of patients was 59 (IQR 48–72; range 28–76) years, and 12 individuals (n = 12, 71%) were male. Common phenotypes were cerebellar ataxia (n = 12, 71%), brainstem encephalitis (n = 12, 71%), opsoclonus-myoclonus syndrome (n = 8, 47%), and limbic encephalitis (n = 3, 18%). Meningitis was observed in one patient. Concurrent antibodies included those against N-methyl-D-aspartate receptor (n = 2), glial fibrillary acidic protein (n = 1), and contactin-associated protein-like 2 (n = 1). MRI was abnormal in 8 cases (47%), showing T2/FLAIR hyperintensity in the rhombencephalon (n = 3, 18%), limbic system (n = 4, 24%), or cerebellar atrophy (n = 2, 12%). Tumors were identified in 10 cases (59%), including seminoma (n = 5, 29%), ovarian teratoma (n = 1, 6%), urological (renal cell carcinoma and urothelial cell carcinoma, both n = 1, 12%), small-cell lung cancer, and carcinoma of unknown primary (all n = 1). Fifteen patients received first-line immunotherapies (88%), and 6 patients also received second-line immunotherapy (35%). Improvement or stabilization was achieved in 10 (7 and 3, respectively, 67%) patients. The median follow-up duration was 20 months (range 1.5–180). Six patients died within the first 12 months, related to encephalitis (n = 3, 18%) or cancer (n = 3, 18%).

**Discussion:**

Anti-KLHL11 encephalitis mainly related to infratentorial encephalitis but can also present as limbic encephalitis and rarely as meningitis. Early diagnosis enables early oncological and immunologic treatment, hopefully improving outcomes.

## Introduction

It has been known for several decades that subacute neurologic conditions can occur as a rare consequence of tumors and the response by the patients' own immune system. These so-called paraneoplastic neurologic syndromes (PNS) often can be identified by specific antibodies, associated with particular clinical phenotypes and tumors, as recently recapitulated in the revised PNS criteria.^1^ In general, most antibodies are aimed against intracellular proteins, which cannot directly interact with their target antigens, and are not considered pathogenic. Nevertheless, these are very relevant biomarkers for a severe disease, and the identification of antibodies will help to identify the relevant tumors and start treatment to halt further progression of neurologic symptoms. Among these recent evolutions, anti–kelch-like protein 11 antibodies (anti-KLHL11-abs) were first described in 13 patients featured by middle-aged males with testicular seminoma and rhombencephalitis in 2019,^2^ potentially defining a new type of PNS (anti-KLHL11 PNS). A broader clinical spectrum of anti-KLHL11 PNS is emerging as more atypical phenotypes are reported.^3-10^ Some had coexisting anti–N-methyl-d-aspartate receptor (NMDAR) antibodies and associated ovarian teratoma, while others had co-occurring anti-Ma2 antibodies and opsoclonus-myoclonus syndrome (OMS). Therefore, the clinical significance of anti-KLHL11-abs still requires further exploration.

So far, over a 100 anti-KLHL11 encephalitis cases were reported worldwide,^11^ but many of them are single case reports, and only 4 case series have been published.^2-4,10^ In this article, we report 17 additional patients and have positioned these along the published cohorts to comprehensively describe the clinical presentation, initial symptoms, radiologic findings, and oncologic associations. While the manifestations, auxiliary examination findings, and tumor correlation of anti-KLHL11 encephalitis received relatively widespread attention and discussion, the therapy response and long-term prognosis are less well studied. To increase clinical recognition, we recorded and describe the reaction to treatment and patients outcome in detail.

## Methods

### Patients

**Figure 1:**
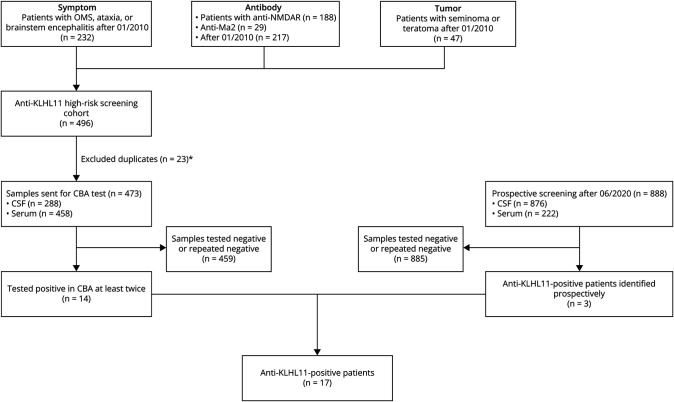
Flowchart of Patient Screening *Duplicates: Four patients occurred in both the “Symptom” and “Antibody” group, 2 patients in both the “Symptom” and “Tumor” groups and 17 patients in both the “Antibody” and “Tumor” group. CBA = cell-based assay; NMDAR = N-methyl-d-aspartate receptor; OMS = opsoclonus-myoclonus syndrome.

We combined a retrospective screening with prospective testing to detect the presence of anti-KLHL11-abs (Figure 1). We selected 458 serum and 288 CSF samples from 473 patients who were referred to Erasmus Medical Center after January 2010 for suspected immune mediated encephalitis, enriched for factors associated with anti-KLHL11 PNS in existing literature: 232 patients had classic PNS, such as OMS, cerebellar ataxia or brainstem encephalitis. One hundred eighty-eight patients were anti-NMDAR positive, and 29 patients were anti-Ma2 positive (antibodies that have previously been described to co-occur with anti-KLHL11-abs). Forty-seven patients had diverse neurologic symptoms in the context of germ cell tumors, including teratoma and seminoma. Prospective screening for anti-KLHL11-abs in a wider perspective without special enrichment are performed as clinical usual practice after June 2020, and a total of 222 serum and 876 CSF samples from 888 patients were tested.

For patients who tested positive from both cohorts, we collected detailed clinical information, including demographic, neurologic, oncologic, and treatment-related data, and discussed them with more than 3 neurologists to confirm the diagnosis. Neurologists that were in charge contacted the anti-KLHL11-abs positive patients to obtain follow-up information and modified Rankin Scale (mRS) scores. Eight of 10 surviving patients (80%) were evaluated at EMC.

### Detection of Anti-KLHL11 Antibodies

The antibodies were detected by a human embryonic kidney 293 (HEK293) cells KLHL11 overexpression cell-based assay (CBA). HEK293 cells were seeded into 2.5% gelatin coated 8-well slides and were transfected with Turbo GFP-tagged (tGFP tagged) KLHL11 plasmid (kindly gifted by Dr. Zekeridou, Mayo Clinic, Rochester, MN) and PEI Max mixture following standard procedures.^12^ Turbo GFP-tagged KLHL11 protein expressing transfected cells were fixated with cold 4% paraformaldehyde at 4°C for 15 minutes, permeabilized with 0.2% Triton for 5 minutes, and blocked with 1% bovine serum albumin (BSA) for 1 hour. Next cells were exposed to patient samples diluted in 1% BSA (serum dilution of 1:100, CSF dilution of 1:2) or a positive control (commercial rabbit anti-KLHL11-ab diluted 1:200 #Atlas Antibodies-HPA054269) for 1 hour and subsequently incubated with corresponding secondary antibodies diluted 1:1,000 (goat anti-human Alexa Fluor 594 #Invitrogen-A11014; goat anti-rabbit Alexa Fluor #Invitrogen-A11012) for 1 hour. Slides were mounted with 4′,6-diamidino-2-phenylindole (DAPI) (Vector laboratories-H1500). Images were taken by ST5LIA_311 Leica Stellaris5 confocal microscope. The results were scored by 2 independent observers, samples exhibiting a bright sparkle fluorescent pattern were repeated, and those remaining to show positivity were recognized as anti-KLHL11-abs positive. In cases with atypical clinical phenotypes, additional tissue staining using immunohistochemistry was performed, which was mandatory to be considered convincing, despite the only moderate reported sensitivity of tissue screening.^2,4^ In total, 16 serum and 13 CSF samples of 16 patients were applied to rat brain sections (eMethods).

To confirm the specificity of our CBA, Western blot (4 serum samples) and immunoblot (all 17 serum samples and 2 available CSF samples from the patients identified by CSF only) were performed (eMethods).

### Statistics

Categorical variables were presented as frequencies (percentage) and compared by the Fisher exact test. Numerical variables were expressed as median (range) and analyzed by Mann-Whitney *U* test. The Kaplan-Meier survival analysis was performed to show the outcome and survival probability changes over time. All data were analyzed using GraphPad Prism 9.0.0.

### Standard Protocol Approvals, Registrations, and Patient Consents

Human biobank approval was provided by the Erasmus Medical Center Institutional Review Board (ACCENT, MEC-2020-0418. Written informed consents were obtained from all surviving patients or authorized care givers. The use of animals was approved by the animal ethics committee (AVD10800202216383).

### Data Availability

All data supporting the findings are reported within this article or available by request from qualified investigators.

## Results

### Patient Screening

As illustrated in the flowchart (Figure 1), 14 patients were identified among the historical cohort of 473 patients and 3 patients were diagnosed with anti-KLHL11 PNS among the prospective cohort of 888 patients, adding up to 17 patients.

**Figure 2:**
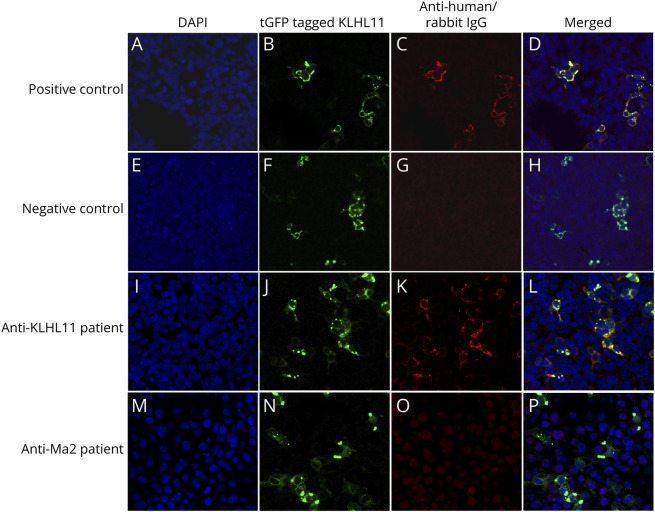
Anti-KLHL11 Antibody (Red) Detected by Cell-Based Assay (A, E, I, M) Nuclei counterstained with DAPI (blue). (B, F, J, N) tGFP tagged KLHL11 expression (green). (C) Reactivity of commercial anti-KLHL11 antibody with HEK293 cells expressing KLHL11 as positive control. (G) No immunoreactivity is observed with a serum negative control. (K) Reactivity of a representative patient's serum. (O) No immunoreactivity is observed with an anti-Ma2+ patient's serum. (D, H, L, P) Merged reactivities of samples, showing colocalization in commercial anti-KLHL11 antibody and anti-KLHL11 patient's serum. Images were captured at ×200 magnification.

We identified 17 patients with positive anti-KLHL11-abs by CBA, 12 were positive in both serum and CSF, 3 only in CSF and for the remaining 2 only serum was available. In all positive patient samples, CBA showed a distinct “cytoplasmic clustered spark-like” fluorescence pattern, colocalizing with the GFP tagged KLHL11 (Figure 2). As for tissue-based assay, only 2 patients showed a positive reaction in both serum and CSF, while single serum staining were recorded in 7 patients. All positive samples showed a perinuclear cytoplasm staining in hippocampus hilus and dentate nuclei of the cerebellum (eFigure 1).

Western blot showed bands at around 80 kDa, in line with the expected size of KLHL11 protein, confirming the specificity of the blots. The immunoblot confirmed the anti-KLHL11 antibody presence in all serum samples of anti-KLHL11 encephalitis patients and 2 CSF samples of the patients identified by CSF only (eFigure 2).

### Clinical Profile

Twelve individuals (71%) in our study were male, a similar proportion as in previous reports 66/82 (80%) (Table 1).^3,4,10^ The median age of patients was 59 years (IQR 48–72; range 28–76 years), slightly older than in previous studies. The most common clinical presentation was cerebellar ataxia (n = 12, 71%) and brainstem encephalitis (n = 12, 71%), followed by OMS (n = 8, 47%), acute vestibulopathy (n = 6, 35%), and limbic encephalitis (n = 3, 18%). These clinical phenotypes are consistent with those described in the US cohort (with a majority having infratentorial symptoms), while cohorts from Spain and France reported higher proportions of limbic encephalitis. Respiratory or consciousness disorder, which can be a sign of severe brainstem involvement, occurred in 4 (24%). The same 4 were those that needed intensive care, and unfortunately, all decreased (median survival 5 months [range 1.5–12 months]). Noteworthily, hearing impairments including hearing loss (n = 2, 12%) and tinnitus (n = 2, 12%) were distinctive symptoms. Prodromal infection (n = 2, 12%) was also observed. The detailed information is presented in eTable 1.

**Table 1:** Clinical Profile of Anti-KLHL11 Positive Patients

Characteristics	Netherlands 2025 N = 17	Spain 2020^4^ N = 32	United States 2020^2,3^ N = 39^a^	France 2021^10^ N = 11
Sex, male (%)	12 (71)	16 (50)	39 (100)	11 (100)
Age, median (IQR; range), y	59 (48–72; 28–76)	28 (\; 9–76)	46 (\; 28–73)	44 (42–51; 35–79)
Clinical syndrome (%)^b^				
Cerebellar ataxia	12 (71)	7 (22)	32 (82)	11 (100)
Acute vestibulopathy	6 (35)	\	21 (54)	8 (73)
OMS	8 (47)	5 (16)	\	\
Brainstem encephalitis	12 (71)	6 (19)	32 (82)	7 (64)
Limbic encephalitis	3 (18)	10 (31)	7 (19)	5 (45)
Hearing impairment	4 (24)	\	29 (74)	5 (45)
Prodromal infection	2 (12)	\	\	\
Tumor (%)	10/17 (59)	23/32 (72)	32/36 (89)	9/11 (82)
Seminoma	3 (18)	4 (13)	21/36 (58)	2 (18)
Burn-out germ cell tumor	2 (12)	0	7/36 (19)	5 (45)
Mixed germ cell tumor	0	3 (9)	2/36 (6)	2 (18)
Teratoma	1^c^ (6)	14 (44)	0	0
SCLC	1 (6)	1 (3)	0	0
Renal cell carcinoma	1 (6)	0	0	0
Urothelial cell carcinoma	1 (6)	0	0	0
Lymph node metastasis of unknown origin	1 (6)	0	0	0
Others^d^	0	1 (3)	2/36 (6)	0
Interval from onset to tumor diagnosis, median (IQR; range), m	6 (3.25–9.5; 0.5–21)	\	\	2 (\; 1–27)
CSF (%)	15/16 (94)		29/34 (85)	11/11 (100)
Pleocytosis	12/16 (75)	\	29/34 (85)	8/11 (73)
Increased protein level	13/15 (87)	\	29/34 (85)	8/11 (73)
Oligoclonal bands	7/10 (70)	\	18/22 (82)	8/9 (89)
Elevated IgG index^e^	4/8 (50)	\	\	\
Other antibodies (%)	4/17 (24)	14/32 (44)		1/11 (9)
Anti-NMDAR +	2 (12)	7 (22)	\	0
Anti-GFAP +	1 (6)	0	\	0
Anti-CASPR2 +	1 (6)	0	\	0
Anti-Ma2+	0	6 (19)	\	1 (9)
Anti-Hu+	0	1 (3)	\	0
MRI abnormalities (%)	8/17 (47)		30/37 (81)	10/11 (91)
T2/FLAIR hyperintensity in brainstem or cerebellum	3 (18)	\	13/37 (35)	4 (36)
T2/FLAIR hyperintensity in supratentorial region	4 (24)	\	15/37 (41)	6 (55)
Cerebellar atrophy	2 (12)	\	6/12 (50)	8 (73)
Outcome measures				
Clinical improvement/stabilization after treatment (%)	10/15 (67)	\	19/33 (58)	4 (36)
Improvement (%)	7/15 (47)	\	9/33 (27)	2 (18)
Stabilization (%)	3/15 (20)	\	10/33 (30)	2 (18)
Death at the last follow-up (%)	7/17 (41)	\	8/33 (24)	3 (27)
mRS at the last follow-up, median (IQR; range)	4 (2–6; 1–6)	\	4 (\; 2–6)	4 (4–6; 3–6)
Follow-up period, median (IQR; range), m	20 (4–47; 1.5–180)	\	30 (\; 2–216)	42 (31–62; 2–126)

Abbreviations: CASPR2 = contactin-associated protein-like 2; FLAIR = fluid-attenuated inversion recovery; GFAP = glial fibrillary acidic protein; IgG = immunoglobulin G; mRS = modified Rankin Scale; NMDAR = N-methyl-d-aspartate receptor; OMS = opsoclonus myoclonus syndrome; SCLC = small-cell lung cancer.

\ = No data available.

a The data from 13 patients reported in 2019^2^ were included in the paper describing 39 patients from the United States in 2020.^3^

b The classification of clinical phenotypes was not exactly similar between cohorts.

c One additional patient had a mediastinal teratoma 40 years ago, without recurrence (despite extensive screening), and his disease was therefore considered as nonparaneoplastic.

d One ovarian carcinoma, one metastatic adenocarcinoma of the lung, and one chronic lymphatic leukemia.

e An IgG index >0.7 was defined as elevated in our study.

**Figure 3:**
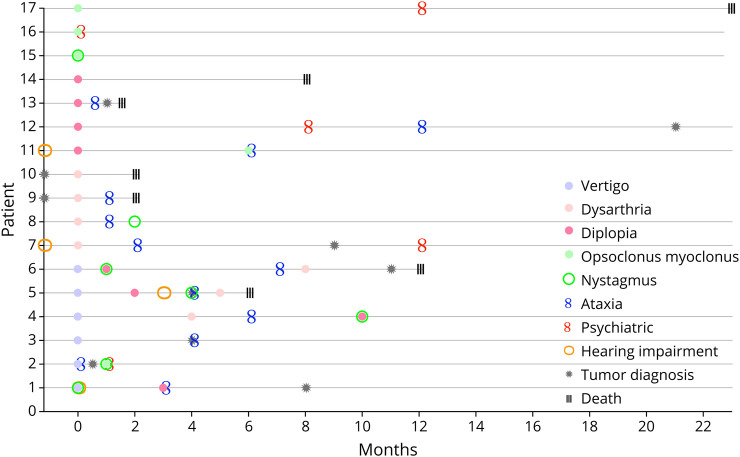
Timeline of Anti-KLHL11 Encephalitis Patients' Initial Symptoms, Tumor Diagnosis and Death Patient numbers correspond to those in eTable 1. *Timing footnotes: Hearing impairment of patient 7 started 30 years earlier, hearing impairment of patient 11 started 15 years earlier, tumor of patient 9 started 1 year earlier, tumor of Patent 10 started 2 years earlier; patient 17 died 9 years afterwards.

Before onset of neurologic symptoms, 2 patients had been diagnosed with tumors. Among the remaining 8 tumor-positive patients, the median interval from onset to tumor discovery was 6 months (IQR 3.25–9.5; range 0.5–21 months). Before onset of neurologic symptoms, 2 patients had experienced impaired hearing. Regarding the other 2 patients with hearing impairment, one developed this symptom together with their neurologic symptoms, while the other developed it 3 months after illness onset. At disease onset, acute vestibulopathy was the most common presentation (n = 6, 35%), 4 of them had acute vertigo as their sole presenting symptom, while 1 had combined nystagmus and hearing impairment and one had simultaneous ataxia. Moreover, acute vestibular symptoms such as vertigo, nausea/vomiting and imbalance appeared exclusively in the initial phase of the disease. Some were transient and paroxysmal, while others persisted for months and developed into a chronic vestibulocerebellar syndrome with prominent ataxia. Thereafter, onset with brainstem involvement was also fairly common. Four patients (24%) had dysarthria, and 4 patients (24%) had diplopia as their only initial symptoms. In addition, the other 3 patients all presented with OMS, accompanied by psychiatric symptoms in one of them (Figure 3).

A previously unreported phenotype was encountered in one of these patients. It was a middle-aged male with acute onset, characterized primarily by persistent fever and decline in consciousness, followed by development of catatonia, rigidity, and dysarthria after 1 week. Neurologic examination revealed meningismus. Meningitis was initially suspected, but CSF analysis only showed slightly increased protein levels and MRI revealed general atrophy without focal lesions. Subsequently, anti-KLHL11-abs were detected in both serum and CSF, and the positivity was confirmed by a cytoplasmic, mostly perinuclear staining pattern on immunohistochemistry (eFigure 1). The patient had been diagnosed with renal cell carcinoma and hepatic metastases 2 years before the onset and tragically died 2 months after onset.

Comparable with previous studies,^3,4,10^ CSF analysis showed inflammatory changes in most cases: leukocyte counts exceeding 5 cells/μL (12/16, 75%), protein 0.45 g/L or greater (13/15, 87%), CSF-specific oligoclonal bands (7/10, 70%), and elevated immunoglobulin G index (4/8, 50%). The median leukocyte count was 18 cells/μL (range 1–89), and the median total protein was 0.84 g/L (range 0.29–1.60) (Table 1).

Four patients had co-occurring antibodies (anti-NMDAR n = 2, antiglial fibrillary acidic protein (anti-GFAP) n = 1, anti–contactin-associated protein-like 2 (anti-CASPR2) n = 1). Three of them showed overlap in the phenotypic spectrum, while one presented as purely anti-NMDAR encephalitis. The patient double positive for anti-KLHL11-abs and anti-GFAP-abs had OMS, ataxia, vertigo, nausea, vomiting, and weight loss, and a teratoma was detected. The patient with concurrent anti-CASPR2-abs presented with OMS, limb tremor and weakness, sweating, seizures, and psychiatric symptoms. The MRI showed mild mesiotemporal T2/FLAIR hyperintensities, consistent with limbic encephalitis, and no tumor was found in this patient. Earlier reported coexisting antibodies include anti-NMDAR, anti-Ma2, and anti-Hu.^4^ Interestingly, no anti-KLHL11 positivity was detected in our anti-Ma2 positive cohort.

**Figure 4:**
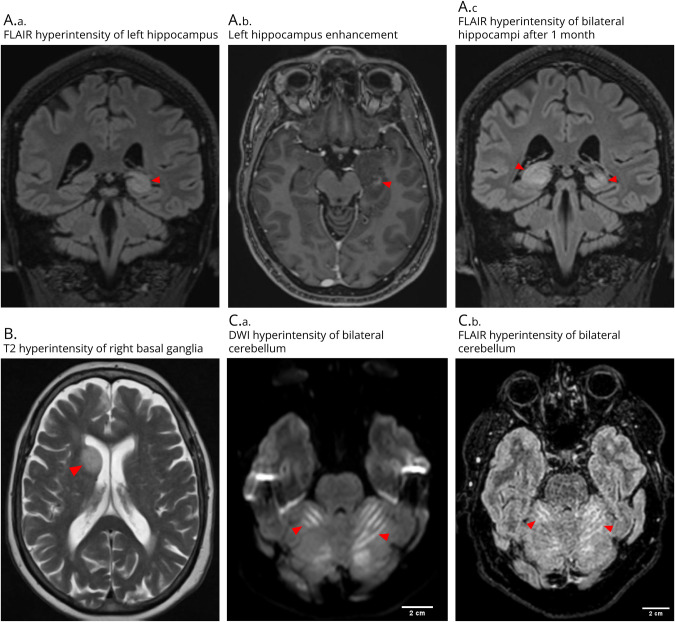
Examples of MRI Abnormalities of Anti-KLHL11 Encephalitis Patients (A.a–A.c) Patient 12: Fluid-attenuated inversion recovery (FLAIR) hyperintensity involving left hippocampus with enhancement on T1 postgadolinium, bilateral lesions were observed at follow-up MRI after 1 month. (B) Patient 14: T2 hyperintensity involving right basal ganglia. (C.a–C.b) Patient 3: Diffusion-weighted imaging (DWI) and FLAIR hyperintensity involving bilateral cerebellum. Patient numbers correspond to those in eTable 1.

Among these 17 cases, more than half (n = 9, 53%) had no abnormalities or only nonspecific changes on brain MRI. MRI abnormalities were less common than in previous cohorts, while the type of abnormalities (when present) were similar.^3,10^ The most common neuroimaging abnormalities were supratentorial T2/FLAIR-weighted hyperintensities (n = 4, 24%). Two patients had hyperintensities in the hippocampus, one of which had bilateral involvement with contrast enhancement on the left side. One patient presented with both supratentorial and infratentorial hyperintense lesions in the right caudate nucleus and left brainstem. The other patient with brainstem lesions showed hyperintensities in the vestibular nucleus, pons, and bilateral middle cerebellar peduncles. Hyperintensities in bilateral cerebellum were observed in one case. Thus, a total of 3 (18%) patients had rhombencephalon lesions. Less commonly, cerebellar atrophy was observed in 2 (12%) patients in the relatively early stage of disease (Figure 4).

### Tumor Association

All patients were screened for cancer and an associated tumor was detected in 10 (59%) patients. The most frequent discovery was testicular cancer (n = 5, 29%), 3 cases with invasive tumor and 2 cases with spontaneously regressed (“burned-out”) testicular germ cell tumor. One patient (6%) had an ovarian teratoma. Two patients (12%) had urological neoplasms: one renal cell carcinoma with hepatic metastases and the other lymph node metastases from urothelial cell carcinoma. One patient had small-cell lung cancer (SCLC) with lymph node and left parotid gland metastasis and another had lymph node metastases from a small cell neuroendocrine carcinoma with unknown primary. Among 5 cases of seminoma, CT failed to detect lesions in 3 out of the 4 examined cases, whereas ultrasonography identified all 4 examined cases, and fluorodeoxyglucose positron emission tomography (FDG-PET) only detected 2 of 5. CT detected all other tumors (the urological tumor cases, SCLC, teratoma, and lymph node metastasis cases). FDG-PET, if performed, confirmed the abnormality in all, but for the teratoma (Table 2).

**Table 2:** Tumor Screening Methods

Tumor (n)	CT (a/b)*	Ultrasound (a/b)*	FDG-PET (a/b)*
Testicular tumors (5)	1/4	4/4	2/5
Teratoma (1)	1/1	0/0	0/0
Urological tumor (2)	2/2	0/0	1/1
SCLC (1)	1/1	0/0	1/1
Lymph node metastasis of unknown origin (1)	1/1	0/0	0/0

Abbreviations: FDG-PET = fluorodeoxyglucose positron emission tomography; SCLC = small-cell lung cancer.

*a: number of patients tested positive; *b: number of patients in whom this test was performed.

### Prognosis and Survival Analysis

Nearly all patients (n = 15, 88%) received one or more of the following first-line immunotherapies: IV immunoglobulin (n = 13, 76%), IV methylprednisolone (n = 12, 71%), and plasma exchange (n = 3, 18%), except for one patient improving spontaneously without any treatment and another patient who died shortly after onset. A subset of 5 patients (29%) received one or more of the following second-line immunosuppressive agents: cyclophosphamide (n = 3, 18%) and rituximab (n = 3, 18%). Acute vestibulopathy (3/6, 50%) and OMS (5/8, 63%) were more frequently responsive to treatment. By contrast, hearing loss (0/4, 0%), bulbar palsy (0/7, 0%), and ophthalmoplegia (2/7, 29%) were largely refractory. As for tumor therapy, apart from the 2 patients diagnosed with burned-out testicular germ cell tumors in whom tumor had regressed spontaneously, the remaining 8 paraneoplastic patients were treated with surgery, chemotherapy, or radiotherapy. Postimmunotherapy (n = 15) clinical improvement was achieved in 7 (47%) and stabilization in 3 (20%), adding up to 10 patients (67%). However, one patient neurologically deteriorated again at 7 months and died at 8 months, but repeat CSF testing for anti-KLHL11 was negative. Another patient had a cerebral hemorrhage at 60 months and died at 108 months.

**Figure 5:**
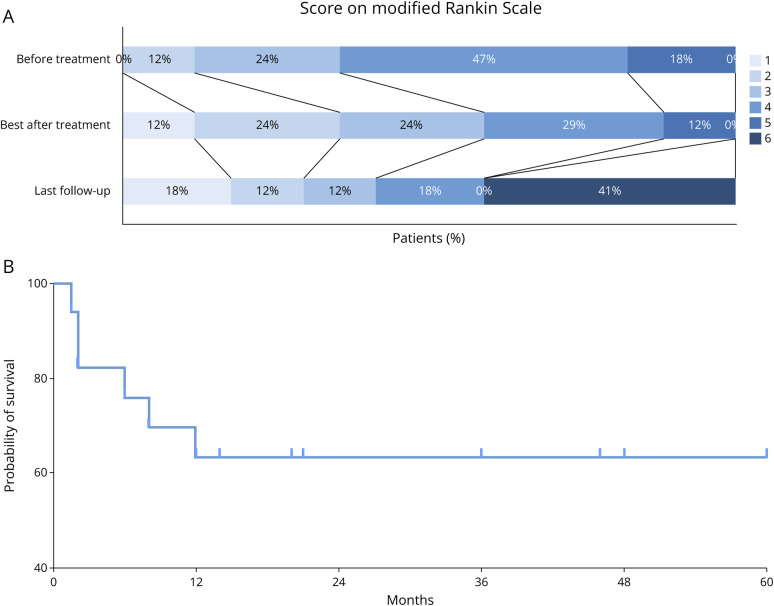
Follow-Up and Outcome of Anti-KLHL11 Encephalitis Patients (A) Distribution of maximum mRS after onset, best mRS achieved after treatment and mRS at the last follow-up. (B) Survival analysis based on Kaplan-Meier curves.

The median follow-up duration of all patients was 20 (IQR 4–47; range 1.5–180) months. The distribution of mRS scores showed a favorable shift of functional outcome after treatment (Figure 5A), proportion of walking independence (mRS ≤3) increased from 36% at the point of worst neurologic function to 60% after treatment, although decreased to 42% at the last follow-up. A total of 7 patients (41%) died during the follow-up period; the median interval was 6 (IQR 2–12; range 1.5–108) months. Three patients (18%) died because of progression of encephalitis and deterioration of neurologic functions. The death of another 3 patients (18%) was related to tumor progression and metastases. The final patient died 108 months after onset due to cerebral hemorrhage (unrelated). Kaplan-Meier curve analysis (Figure 5B) indicated that most deaths (6/7, 86%) occurred within the first year, after which the survival rate remained relatively stable.

## Discussion

In this study, the clinical features of the Dutch cohort of anti-KLHL11 encephalitis patients were investigated in detail, which provided important information for clinical practice. First, the analysis of prognostic and long-term follow-up results suggests that the effect of immunotherapy is better than previously reported. Second, broader oncologic associations were found, especially with urinary tract tumors, which are not typically associated with PNS. Finally, novel phenotypes and coexisting antibodies were identified, expanding the disease spectrum.

Two-thirds of patients benefited from immunotherapy, including improvement in half of the treated cases and stabilization in some others. The proportion of patients improved exceeded expectations as previous reports mentioned 27% and 18% benefited from immunotherapy, while stabilization was comparable with previous reports (30% and 18%, respectively).^3,10^ The more favorable treatment response observed in our cohort may be attributed to population differences, such as milder baseline neurologic dysfunction, exemplified by a lower median mRS in our study. Furthermore, differences in the assessment of treatment response might also be a contributing factor. We recorded performance and fluctuations over time, pursuing for long-term follow-up, and compared best mRS achieved after treatment to pretreatment status, in addition to mRS at the last follow-up. All previous studies used mRS to evaluate patients' neurologic function as well but compared only mRS at the last follow-up to pretreatment status. Besides, differences due to limited sample size should also be taken into consideration. Most classic PNS, such as anti-Hu encephalomyelitis, tend to respond poorly to immunologic and/or oncological therapies,^13^ while antibodies against extracellular antigens generally show better outcomes.^14^ Our findings suggest that the clinical outcome of anti-KLHL11 encephalitis is somewhat different, showing poor outcome in part of the patients while showing a better response to treatment in a considerable part of the patients. This might be related to variations in sites of involvement and initial phenotypes: patients presenting with vertigo or OMS tend to have a more favorable prognosis, whereas those who developed bulbar palsy at an early stage were associated with a more aggressive course. Similarly, our survival analysis suggests that mortality primarily occurs within the first year; when patients survived this critical stage they tended to remain stable and did relatively well over the long time, indicating that the acute phase of this disease is the most perilous, which underscores the necessity and value of rigor, speed, and intensive treatments. Prompt and individualized immunotherapy should be considered once anti-KLHL11 encephalitis is suspected, and an infectious cause has been ruled out. Previous studies also showed that patients in whom testicular cancer was diagnosed and treated had a more favorable clinical outcome. As cancer is the source of autoantigen-driving autoimmune response, its surveillance and management are of crucial importance.^3^ This highlights the importance of dedicated tumor screening.

In our cohort, tumors are found in approximately two-thirds of the patients, including some novel tumor associations. Next to the classically encountered seminomas and teratomas, some atypical malignancies including SCLC, lung adenocarcinoma and chronic lymphocytic leukemia were previously reported in anti-KLHL11.^3,4^ In addition, we report urinary tract tumors in 2 patients, an association not described before. One case involved a renal cell carcinoma with hepatic metastasis and the other had lymph node metastases from urothelial cell carcinoma. However, given the lack of KLHL11 expression data in tumor tissues, fortuitous associations cannot be ruled out. Villagrán-García et al.^15^ found that high-risk antibodies were infrequent in renal cell carcinoma-associated PNS. Thus, the relation between PNS and renal cell carcinoma should be interpreted with caution and warrants further research. However, Otis et al.^16^ found that most urinary cancers linked PNS presented with high-risk phenotypes and 10/25 of suspected urothelial cancer-associated PNS had high-risk antibodies, with anti-Hu, anti-Yo, anti-Ri being the most common ones, strengthening the paraneoplastic nature of this association. It needs mentioning that renal cell carcinomas were not included in this last study. Therefore, patients presenting with new-onset brainstem cerebellar syndromes should also undergo detailed assessment for urinary tract tumors, dedicated screening such as urinary tract ultrasound, abdominal and pelvic CT/MRI, and even FDG-PET is worthwhile in these highly tumor-related diseases, to optimize the sensitivity. Consistent with previous research,^3,4,10,17^ anti-KLHL11 encephalitis patients are highly likely to have underlying tumors, and seminoma are the most common neoplasms. However, in clinical practice, smaller ones are easily missed with only routine tests,^3,18^ especially the burned-out testicular tumors.^19^ In our study, neither CT nor FDG-PET, usually considered a comprehensive screening tool in clinical practice, were very sensitive to detect testicular seminomas. As FDG-PET measures glucose metabolism, “cold” tumors are less well detected.^1,20^ Ultrasound was more sensitive to detect abnormalities, but experience and a high level of vigilance and suspicion were indispensable. As the prior chance was very high, even small abnormalities (calcifications) should be considered as a potential tumor, necessitating tissue confirmation.^18,21^

From a clinical perspective, the results herein suggest a rather homogeneous phenotype, as anti-KLHL11 encephalitis often presents with infratentorial symptoms, including brainstem and cerebellar involvement and vestibulocochlear neuropathy.^2^ However, additional clinical phenotypes were observed, including approximately one in 5 patients presenting with limbic encephalitis. This percentage is lower than in some previous studies, possibly reflecting certain referral patterns or interests of specific sites.^4,10^ Furthermore, we reported a new phenotype, with manifestations mimicking meningitis and catatonia, which are more associated with antibodies to GFAP^22^ and NMDAR,^23,24^ respectively, which have not been reported in anti-KLHL11 encephalitis before. However, the patient with a meningitis-like presentation and catatonia did not have concurrent anti-GFAP nor anti-NMDAR antibodies. Nonetheless, considering the limited clinical information and rapid death of the patient limiting the amount of ancillary testing, we cannot be certain that all symptoms can be attributed to the diagnosis of anti-KLHL11 encephalitis in this patient. Some previously reported phenotypes such as primary neurodegenerative disorders^10^ and longitudinal extensive myelitis^11^ were not observed in our study.

The limitations of our study include the small sample size, which is largely due to the rarity of this disease,^17,25^ restricting the application of statistical analysis and generalizability of our findings. The low incidence of this condition makes prospective recruitment challenging, so a retrospective design was conducted, which led to incomplete data collection and potential referral bias toward more severe cases. Furthermore, as anti-KLHL11-abs have been identified relatively recent and there are currently no clinical consensus or guidelines on treatment, there were variations in treatment strategies among patients, which may introduce confounding factors and make it difficult to determine the true efficacy of specific interventions. Well-designed, multicenter, large-cohort, prospective and long-term follow-up studies are needed to confirm the broader oncological associations, treatment effect, and prognosis.

In conclusion, anti-KLHL11 encephalitis should be considered specifically but not only in patients exhibiting infratentorial symptoms, as these clinical signs are most prominent in this disease. Importantly, auxiliary test results are not very specific and might be less suspicious for encephalitis. In young men/women with atypical phenotypes, especially if they have imaging abnormalities in testes or ovaries, the screening for anti-KLHL11-antibodies should not be neglected. Dedicated tumor screening including reproductive and urinary systems is recommended, which is essential for reducing missed tumor diagnosis and delayed treatment. Although part of the patients showed poor treatment response, most patients did respond to immunosuppressive treatment and, if applicable, in combination with tumor treatment. Early and proactive therapy is recommended and can benefit a considerable part of patients to achieve better long-term outcomes.

## Glossary

BSA: bovine serum albumin
CASPR2: contactin-associated protein-like 2
CBA: cell-based assay
FDG-PET: fluorodeoxyglucose positron emission tomography
FLAIR: fluid-attenuated inversion recovery
GFAP: glial fibrillary acidic protein
IgG: immunoglobulin G
KLHL11: Kelch-like protein 11
mRS: modified Rankin Scale
NMDAR: N-methyl-d-aspartate receptor
OMS: opsoclonus myoclonus syndrome
PNS: paraneoplastic neurologic syndromes
SCLC: small-cell lung cancer
